# Ultra-Processed Foods—Dietary Foe or Potential Ally?

**DOI:** 10.3390/nu16071013

**Published:** 2024-03-30

**Authors:** Sabrina Nees, Tymofiy Lutsiv, Henry J. Thompson

**Affiliations:** 1 Graduate Program in Horticulture and Human Health, Colorado State University, Fort Collins, CO 80523, USA; sabrina.khalid@colostate.edu; 2 Cancer Prevention Laboratory, Colorado State University, Fort Collins, CO 80523, USA; tymofiy.lutsiv@colostate.edu; 3 Graduate Program in Cell and Molecular Biology, Colorado State University, Fort Collins, CO 80523, USA

**Keywords:** non-communicable diseases, food processing, nutritional quality, nutrient density, ultra-processed foods

## Abstract

The prevalence of non-communicable diseases (NCDs) has steadily increased in the United States. Health experts attribute the increasing prevalence of NCDs, in part, to the consumption of ultra-processed foods (UPFs) based on epidemiological observations. However, no definitive evidence of causality has been established. Consequently, there is an ongoing debate over whether adverse health outcomes may be due to the low nutrient density per kilocalorie, the processing techniques used during the production of UPFs, taste preference-driven overconsumption of calories, or unidentified factors. Recognizing that “the science is not settled,” we propose an investigative process in this narrative review to move the field beyond current controversies and potentially identify the basis of causality. Since many consumers depend on UPFs due to their shelf stability, affordability, availability, ease of use, and safety from pathogens, we also suggest a paradigm for guiding both the formulation of UPFs by food designers and the selection of UPFs by consumers.

## 1. Introduction

Western-type dietary patterns have expanded to all parts of the world. Many countries at various stages of economic development are adopting a ”Western staple” into their diets—ultra-processed food (UPF). UPF products, originally classified as such because they were designed to be highly palatable and ready-to-eat, energy-dense foods high in sugar, salt, and saturated fats, have evolved in their formulation to create a complex landscape that makes classification difficult. While many UPFs lack health-essential micronutrients, phytochemicals, and dietary fiber [1,2], currently available UPFs can be combined to create meal plans with a higher Healthy Eating Index (HEI) than is commonly consumed in the United States [3]. However, epidemiological evidence implicates UPFs in the etiology of non-communicable diseases (also referred to as chronic diseases), such as obesity, type 2 diabetes, cardiovascular disease, and certain types of cancer. This is plausible since these food products contribute ~60% of the total energy consumed per day in the United States in various demographics [4,5]. Consequently, a “debate” about why UPF consumption is linked to NCD risk has emerged. While some scientists argue UPFs are “industrial formulations” that adversely affect health due to their content of salt, sugar, and oils at the expense of nutrients, health-beneficial phytochemicals, and dietary fiber, other investigators posit that detrimental effects are attributable to substances not generated during food preparation at home or used in culinary preparations: in particular, additives that mimic the sensorial qualities of minimally processed foods and their culinary preparations [6]. Whether taste preference-driven overconsumption of calories relative to energy need, which could be due to both UPF composition and non-culinary additives that many products contain, which results in obesity, is the driver that increases risk for other chronic diseases also merits consideration. Inevitably, this line of questioning has prompted discussions in several countries on whether to ban UPFs from the food system or whether UPFs can be reformulated to improve health outcomes while remaining affordable, convenient, safe, and shelf-stable. In this narrative review, food classification systems are first reviewed, providing a foundation for a research construct to advance the field beyond current controversies. In addition, a paradigm is considered for guiding both the formulation of UPFs by food designers and the selection of UPFs by consumers.

## 2. Food Classification Systems

**Figure 1:**
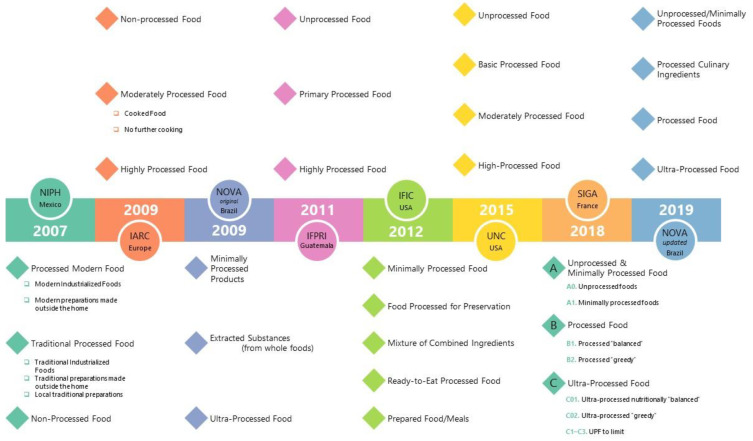
The timeline and summary of primary UPF food classification systems.

Food classification systems are intended to categorize foods by identifying, naming, and defining foods according to shared characteristics. Many countries engage in this process to provide dietary guidance to the population; however, most systems are based on culinary definitions of foods and are designed to meet the recognized nutritional needs of the populations for which they are intended, e.g., the US Dietary Guidelines [7]. On the other hand, concerned about the impact of food processing on human health, scientists have proposed an alternative classification schema. Moreover, as food processing has increased in complexity, several classification systems have emerged. De Araújo et al. (2022) posited that the importance of developing new classification systems is that they become the basis for epidemiological and experimental investigations that can inform nutritional guidelines and nutrition policy [8]. Sadler et al. (2021) state that classification systems based on food processing are intended to examine the link between the nutritional quality of industrial products and the risk of disease [9]. The first food classification system focusing on food processing techniques was created in 2007 by the National Institute of Public Health (NIPH) of Mexico [8]. The ones to follow that distinguish between processed and ultra-processed products include the IARC-Europe, NOVA-Brazil, IFPRI-Guatemala, IFIC-USA, UNC-USA, and SIGA-France (Figure 1), as discussed in [8,10,11].

## 3. The NOVA Classification System

Given that multiple global organizations use the NOVA classification system to inform nutritional policy and that it has become the framework prompting the development of alternative classification systems, we will focus on it to exemplify the nature of concerns with the current classification schema. In the original publication, Monteiro et al. (2009) outlined three food categories in terms of their type, degree, and purpose of processing: minimally processed products, extracted [from whole foods] substances, and UPFs [12]. The minimally processed group includes “whole foods” that have undergone processing but have not been significantly altered in terms of nutritional value. The second group, extracted substances, are products not consumed by themselves and used in “domestic preparation” for dishes made up of “fresh” or minimally processed foods. The last group, UPFs, are produced using food products from Group 2 as raw materials and other processing methods/materials that make them more “edible, palatable, and habit-forming.”

In the most recent update of the NOVA system [13], food groups are categorized based on the extent and purpose of processing (Figure 1). Group 1 includes unprocessed and minimally processed foods to extend the shelf life or make preparation easier (e.g., crushing, drying, freezing, pasteurizing, etc.) without the addition of other ingredients. Group 2 comprises processed culinary ingredients obtained from Group 1 or directly from nature (oils, fats, sugar, and salt), which are then used in the “preparation, seasoning, and cooking of Group 1 foods”. Processing techniques in this group include pressing, centrifuging, refining, extracting, or mining. Processed products of Group 3 are made by adding products from Group 2 to Group 1 via implementing processing methods, such as canning, bottling, or fermentation. These processes aim to “increase durability” and transform the sensory qualities of foods. Lastly, Group 4 is the UPF category, defined as “formulations of ingredients, mostly of exclusive industrial use, that result from a series of industrial processes.” The authors further explain that the production process of UPFs often begins with the fractionation of whole foods into specific substances (oil, sugar, fat, proteins, fiber, starches) that are obtained from “high-yield plant foods” (corn, wheat, soya, cane, or beet) or from the “grinding/pureeing of animal carcasses from intensive livestock farming.” Following this step, foods are subjected to chemical modifications, additional industrial processes of unmodified and modified foods (extrusion, molding, pre-frying), and the addition of additives (colors, flavors, emulsifiers, etc.) that enhance the sensorial qualities and encourage overconsumption. The authors conjecture that the nature of ingredients and processes used in producing UPFs and their subsequent displacement of unprocessed or minimally processed foods make these products unhealthy.

**Figure 2:**
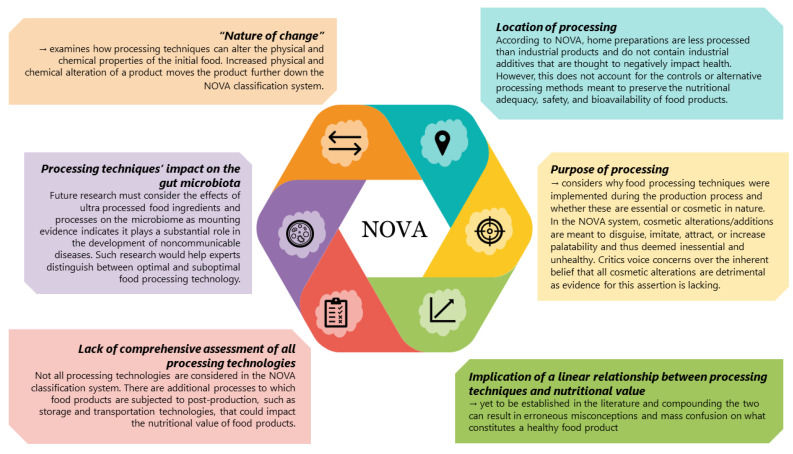
Notable criticisms of the NOVA and by extension other food classification systems [8,9,11,14,18,19].

Despite the prominence of the NOVA system, numerous researchers have challenged many aspects, as summarized in Figure 2 [8,14,15]. Rapid strides are being made in alternative food classification approaches through machine learning and artificial intelligence algorithms [16]. Menichetti et al. (2023) introduced a machine learning algorithm that can indicate the degree of processing for many food products on the market using the product’s nutrition facts label and compare these data to the nutrient content of the unprocessed constituents of the food product [17]. They assert that their technology named *FoodProX* indicates the level of chemical and physical alterations in processed food products and enables them to quantify the diet quality of individual consumers. This work distinguishes itself from other food classification efforts by linking an individual’s dietary quality score to biomarkers and endpoints related to NCD risk.

## 4. Investigating Causality

**Figure 3:**
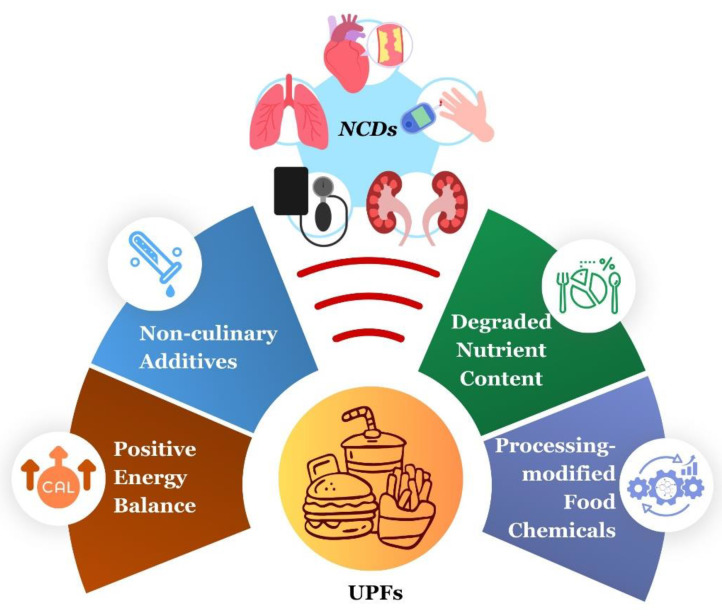
Factors that may account for the effects of ultra-processed foods (UPFs) on the risk of non-communicable diseases (NCDs).

Causal evidence is lacking to substantiate a direct effect of UPFs on consumer health. Based on the food classification review presented above and the data analyses presented in [17], areas for investigation are divided into four complementary categories that are not mutually exclusive. They are energy balance, nutrient density, food processing, and use of non-culinary additives (Figure 3):

❖ ***Energy Balance.*** Over recent decades, numerous studies have found positive associations between UPF consumption and obesity risk [16,18,20,21,22,23]. In a prospective cohort study, Beslay et al. (2020) reported a positive correlation between UPF consumption and obesity [24]. Participants with a higher proportion of UPFs in their diet had a higher risk of obesity (hazard ratio (HR) = 1.09 [1.05–1.13], *p* < 0.001). In a randomized controlled trial conducted by Hall et al. (2019), individuals were presented with processed and unprocessed diets matched in calories, nutrients, and energy density, and the caloric intake was higher on the processed diet (UPF diet 508 ± 106 kcal/day higher, *p* = 0.0001), although there were limitations in the matching of food volume and fiber type and amount between groups [25,26]. A cross-sectional study by Monteiro et al. (2018) found that increased household availability of UPFs is positively associated with the prevalence of obesity, noting that with each percentage point increase in energy share of UPF products, there is potentially an increase of 0.25 percentage points in obesity prevalence [27]. In a Brazilian longitudinal study from 2008 to 2010 [23], Silva et al. (2018) observed higher BMIs in those who habitually consumed larger amounts of UPFs in their diets with those consuming the highest amount of UPFs having a mean BMI 0.80 kg/m^2^ higher (95% CI 0·53, 1·07) than individuals who consumed the least amount of UPFs in their diets. Thus, epidemiological evidence signals an association between increased UPF consumption and obesity, which is considered a gateway disease to other NCDs. However, using a machine learning algorithm to classify the degree of processing for many food products on the market, Menichetti et al. (2023) found positive associations between UPF consumption and NCD-related outcomes despite controlling for body mass index and caloric intake [17]. This suggests that positive energy balance may not be an obligatory driver of causality.
❖ ***Nutrient Content.*** While early work on UPFs focused on their content of refined carbohydrates, saturated fats, and salt, this is no longer an obligatory attribute of UPFs. There are currently nutrient-dense UPFs on the market that refute the hypothesized linear relationship between nutritional value and processing techniques. Notably, a proof-of-concept study, conducted by Hess et al. (2023), illustrated a menu composed of 91% kcal from UPFs that complied with recommendations from the 2020 Dietary Guidelines for Americans and had a Healthy Eating Index-2015 score of 86 out of a possible 100 points [3]. This study demonstrates that UPF products that are nutritionally adequate exist, although whether or not they affect the risk of NCDs is not yet known. Relative to the role of poor nutritional status per se in NCD pathogenesis, the evidence is mixed, which weakens arguments that inadequate nutrient intake is a driver of NCD risk. Rather, poor nutritional status may serve as a biomarker that other causal processes are in play. It can be argued that the findings from Menichetti et al. (2023) support the biomarker hypothesis [17].
❖ ***Food Processing and Non-Culinary Additives.*** Food processing techniques implicated in NCD risk include hydrogenation, extrusion, pre-frying and/or the addition of colorants, emulsifiers, and preservatives. It has been hypothesized that the way foods are “manufactured” relative to their capacity to alter the gut microbiome directly affects metabolic diseases through alterations in the immunity–inflammation axis [19]. The central argument is that UPFs are the actual drivers of the Western dietary pattern’s effect on NCDs. Food processing alterations fall into four categories that merit investigation: (1) consumption of refined macronutrients that are highly digestible, (2) effects of additives (emulsifiers and non-caloric artificial sweeteners), a proposition that has been challenged [28,29], (3) microbial products formed during food handling and processing before a food is consumed (although this can also be a problem, with minimally processed or home-processed foods that are the sources of many more cases of foodborne illness), and (4) novel chemicals formed in a food during processing, e.g., acrylamides and advanced glycation end products, which notably can also be formed during preparation of foods in the home environment.

**Figure 4:**
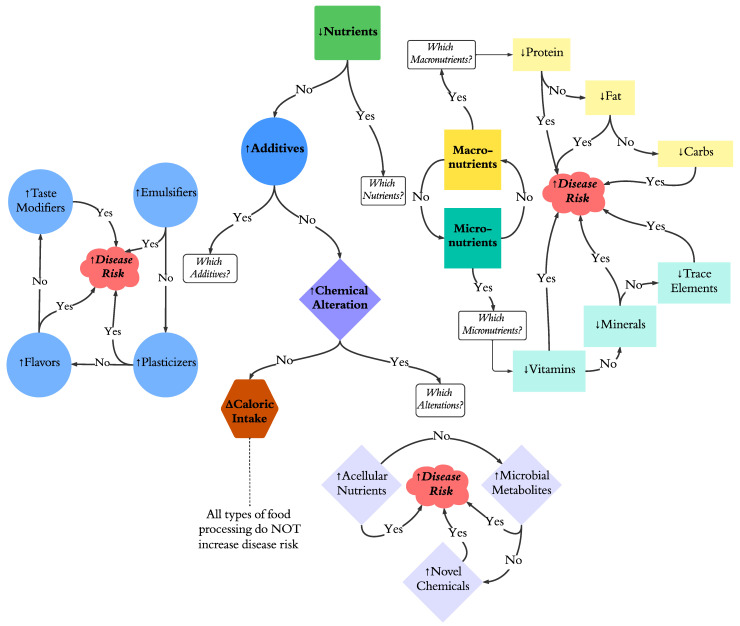
A decision tree to distinguish causal from bystander effects of UPF factors on health-related outcomes. Symbols indicate increase (↑), decrease (↓), or any change (Δ).

The following decision tree is proposed as a conceptual framework to investigate these factors as determinants of causality for the health outcomes associated with UPF consumption (Figure 4). The decision tree does not presume that the factors listed will be determined to be causal agents. If no factor is demonstrated to increase NCD risk, then the arguments that the UPF–NCD risk axis is not coincidental are weakened. We chose the decision tree approach since decision tree methodology is a foundational tool in machine learning, as reported in [17]. It is applied here in order to facilitate the development of a sequential series of falsifiable hypotheses that will help narrow and focus mechanistic inquiry.

The decision tree schema defines a set of experiments to determine whether each factor presented in Figure 3 has no impact or exerts causal or permissive effects in terms of increasing NCD risk. These factors would be sequentially studied. Given the number of variables that need to be examined to make progress, we recommend the use of preclinical models validated for NCDs. One such model in the mouse has the development of hepatic steatosis as the disease endpoint. The rationale for using this model hinges on the short time frame for the development of detectable steatosis and the ability to noninvasively screen for this disease endpoint. Purified diet formulations in combination with human dietary cuisines formulated with UPFs can be used to systematically investigate each UPC factor as hypothesis testing progresses through the decision tree matrix (Figure 4).

## 5. UPF Trends

Despite the ambiguity and lack of consensus regarding UPFs classification and their contribution to NCD risk, it is essential to emphasize their practical importance in the global food system. As an exemplar, consider the United States of America, where UPFs contribute more than half of the population’s caloric intake [4,5,6,30,31,32]. Juul et al. (2022) corroborate these data by analyzing nine cross-sectional waves of the NHANES dietary trends according to processing level from 2001 to 2002 to 2017 to 2018 [33]. UPF consumption increased modestly from 55.2% in 2009 to 57% in 2018. However, more insightful are the trends in consumption across different food categories. Particularly notable were the increases observed in cakes, cookies, and pies, reconstituted meat and fish products, salty snack foods, sandwiches and hamburgers, sauces, sweet snack food, confectionary, and instant/canned soups. Concomitantly, consumption decreased in the following other categories: bread, soda, noncarbonated sweet drinks, breakfast cereal, dairy-based drinks, frozen/shelf-stable meals, ultra-processed potato products, and ice cream. Of these displaced foods, many have been associated with health benefits [34,35]. The overall increase in UPF consumption is significant as it signals the displacement of health benefit-associated UPFs as well as unprocessed/minimally processed foods, which are linked to improved health outcomes [36]. Additionally, among different age groups, slight decreases in UPF intake were observed as follows: 36.9% in 2009–2010 to 36.3% in 2017–2018 for adults ages 20–39 and 38.5% in 2009–2010 to 34.6% in 2017–2018 for 40–59-year-old adults. However, adults 60 years of age and over have steadily increased their consumption of UPFs from 24.5% in 2009–2010 to 29.1% in 2017–2018. Potential explanations for this increase were speculated to be isolation, convenience, economic feasibility, and adverse health behaviors. And in many cases, it is not adverse health behaviors but rather health conditions that impede an elderly person’s ability to acquire, prepare, and afford alternate choices. Ong et al. (2016) also report that older adults living alone are susceptible to poorer health practices like alcohol consumption, tobacco use, physical inactivity, and deprived nutrition [37].

## 6. Moving Forward: Ban or Reformulate UPFs?

The creators of NOVA recommend limiting the intake of UPFs. In a 2019 Food and Agricultural Organization of the United Nations report, Monteiro et al. (2019) highlight policies, government-led initiatives, and recommendations for Latin America [13]:

❖ Promote healthy eating and food environments according to “national circumstances”.
❖ Counteract the displacement of “fresh”/minimally processed foods by UPFs via preserving food systems, supporting family farmers, promoting healthy food preparation, and cooking in public institutions, such as schools.
❖ Create public health policies and market incentives to make unprocessed/minimally processed food more valued, available, and affordable.
❖ Create statutory regulatory measures for UPFs that have little nutritional value.

While this advice is reasonable and highlights the importance of making health-enhancing food affordable and accessible, the recommendations overlook notable attributes of UPFs that make them popular among consumers, i.e., palatability, convenience, safety, and shelf stability. Should policies to limit UPF consumption be enacted, these could significantly increase food insecurity, particularly among vulnerable demographics, such as older adults [32]. An alternative and timely strategy is the reformulation of UPFs. In a cross-sectional analysis of 24-h dietary recall data following directions in dietary macronutrient intake and diet quality among U.S. adults from 1999 to 2016, it was determined that U.S. residents have begun to favor high-quality carbohydrates, plant-based protein, and polyunsaturated fats [38]. Nevertheless, the same study found that U.S. residents continue to consume large amounts of low-quality carbohydrates and saturated fat, indicating that despite notable shifts toward healthier, more nutrient-dense food sources, poor dietary behaviors persist, reflecting the situation’s complexity.

One approach to reformulation that is consistent with the United Nations’s report is known as whole-food formulation. Complete-food formulation is meant to replace “processed, refined, and reconstituted” ingredients with “intact or minimally processed ingredients” [39]. This practice encourages the development of new innovative products rather than reforming older products. Adams et al. (2020) suggest that whole-food formulation is viable if agricultural and economic incentives change from its current model [40]. The authors suggest increasing taxes and decreasing subsidies for UPFs while increasing subsidies for unprocessed and minimally processed products. The definitions would need clarification before implementing such policies; however, the proposal merits more consideration. Moreover, this incentive model would decrease prices for minimally processed products, which is currently an obstacle for a notable portion of the U.S. population. Reformulation and whole-food formulations should be encouraged as each can provide healthier processed options than those currently on the market.

## 7. A Working Model to Guide Product Development and Consumer Choices

A potential model that could be used to advance UPF formulation and to guide human consumption behaviors is the military’s ready-to-eat meals (MREs)—individual rations produced according to strict nutritional standards meant to maximize the nutritional value of foods provided to military personnel and maintain their performance. The rationale for using the MRE framework of the U.S. Army Research Institute of Environmental Medicine (USARIEM) is that the guidelines for ration development are evidence-based nutritional approaches used to promote and support healthy and adequate dietary intake [41]. The nutritional standards for military feeding are adapted from the Dietary Guidelines for Americans and the Food and Nutrition Board (FNB) Dietary Reference Intakes (DRIs) for both macronutrients and micronutrients, respectively. In particular, military DRIs are identical to the references above; however, these are subject to adjustments depending on intrinsic and extrinsic factors, such as gender, body size, physical activity level, environment, clothing and equipment, terrain, and metabolic adjustments, that can alter energy and nutrient requirements [42].

**Figure 5:**
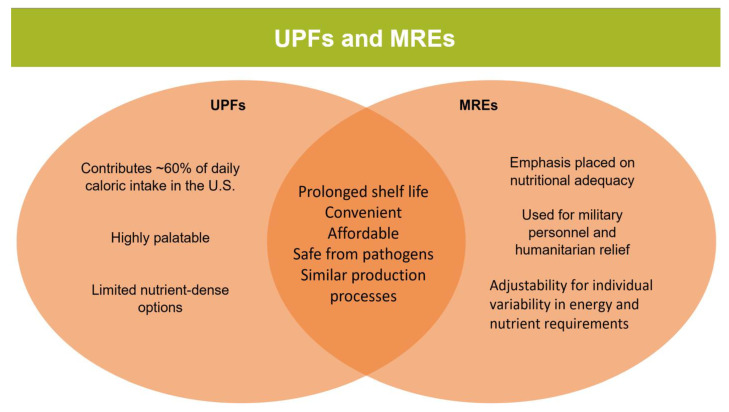
Differences and similarities in UPFs and MREs.

McClung et al. (2020) specify that MREs are made of real food items that are fortified and packaged in a way that ensures the contents within meet nutritional requirements throughout their 3-year shelf life [43]. It is noteworthy that since they are fortified and have more than five ingredients, they would be considered a UPF category in the NOVA system. These meals include an entrée, side, fruit, snack, dessert, beverage, and condiments, all of which can be adjusted for different calorie levels depending on the needs of the consumer. In addition to nutritional adequacy and prolonged shelf life, MRE food technologists also focus on convenience, adjustability for individual variability, food quality, and palatability. Notably, most of these attributes mirror the focus of UPF producers. Moreover, some of the food processing technologies are similar to those of UPFs, such as retort thermal processing, where food is sealed in an airtight container and subsequently heated under pressure to inactivate microbes that cause food spoilage [44]. However, like the food industry, current research efforts are prioritizing nonthermal, low-thermal, and advanced thermal processes, which are noted to be less harmful than conventional thermal processes. The similarities between the two sectors are such that collaboration would result in mutual gains for both industries while simultaneously advancing the health of their consumers (Figure 5).

## 8. Limitations

This evaluation was a conservative analysis of the available literature on UPFs, MREs, and food classification systems. The work is conducted with a U.S.-centric focus, which could potentially underestimate the full impact of UPFs and their proposed reformulation in other countries. Furthermore, the concept of implementing a U.S. military meal development model with rigorous nutrient standards and guidelines within the food industry and public health sectors is a novel idea. However, it requires further investigation since MREs are also criticized relative to culinary appeal, high content of sodium, and bulky packaging.

## 9. Summary

Ultra-processed food products are significantly misunderstood. While associated with negative health outcomes, causal evidence is lacking to substantiate a direct relationship between the extent of processing and the health status of consumers. Moreover, food classification systems, such as NOVA, lack appropriate levels of standardization, which results in vague, inconsistent, and potentially misleading food categorizations. This situation understates the importance of UPFs as these products are deeply entrenched within our global food system and deter food insecurity due to their many positive attributes. Hence, their classification requires nuanced approaches and multi-sector collaboration to provide scientifically sound justification and clear definitions for food processing concepts.

Notably, rapid strides are being made in this regard through the use of machine learning and artificial intelligence algorithms [17]. Menchetti et al. (2023) introduced a machine learning algorithm that can indicate the degree of processing for many food products on the market using the product’s nutrition facts label. They assert that their technology, named *FoodProX,* indicates the level of chemical and physical alterations in processed food products and enables them to quantify the diet quality of individual consumers who use their technology. However, as highly personalized and targeted technology emerges, caution must be exercised to limit potential bias. The nature of any algorithm output is dependent on the information given during its development. Therefore, it is necessary to have a consensus on food categories and definitions to deter such programmer bias.

Technology will also play a pivotal role in the future development of ultra-processed food products because, despite disagreements on UPF reformulation and whole-food formulation, ultra-processed food products will continue to play a major role in our food system. This situation calls for a renewed production process that enables the food industry, in collaboration with other sectors, to produce nutrient-dense processed food products.

Future research on ultra-processed foods should focus on three key points. Firstly, researchers must identify the biochemical mechanisms affected by ultra-processed food consumption and their association with chronic disease risk. Secondly, experts should leverage innovative technology to develop objective machine learning algorithms to create food classification frameworks that accurately determine the nature of food components as well as the degree of processing of a food product and relative to the body of literature that assesses their health implications for consumers. How foods are then labeled must also be reconsidered. Lastly, food industry professionals should consider incorporating military MRE formulation standards for future ultra-processed food reformulation and whole-food formulation to create health-enhancing products for consumers.

Overall, epidemiological evidence associates UPFs with adverse health outcomes. Chronic consumption of UPFs is linked to an increased risk of non-communicable, diet-related diseases, such as obesity, type 2 diabetes, cardiovascular disease, certain types of cancer, and metabolic illnesses. However, the specific attributes of UPFs that play causal roles in disease pathogenesis have not been identified. We outline a decision tree approach to facilitate mechanistic research. Moreover, since UPFs are ubiquitous in the diets of most consumers, we propose the use of the criteria the military has used to formulate MREs in order to (1) identify currently available UPFs that can be combined to provide nutritionally adequate cycle menus, (2) flag UPFs that should be reformulated, and (3) develop a new generation of UPFs that uses minimally processed whole-food ingredients. If used wisely, UPFs can be a significant resource to optimize human health and guarantee future food security, and not represent a dietary foe as they are frequently portrayed.

## Data Availability

Narrative review: Not applicable.

## References

[1] Martini D., Godos J., Bonaccio M., Vitaglione P., Grosso G. (2021). Ultra-Processed Foods and Nutritional Dietary Profile: A Meta-Analysis of Nationally Representative Samples. Nutrients.

[2] Temple N.J. (2022). The Origins of the Obesity Epidemic in the USA-Lessons for Today. Nutrients.

[3] Hess J.M., Comeau M.E., Casperson S., Slavin J.L., Johnson G.H., Messina M., Raatz S., Scheett A.J., Bodensteiner A., Palmer D.G. (2023). Dietary Guidelines Meet NOVA: Developing a Menu for A Healthy Dietary Pattern Using Ultra-Processed Foods. J. Nutr..

[4] Gupta S., Hawk T., Aggarwal A., Drewnowski A. (2019). Characterizing Ultra-Processed Foods by Energy Density, Nutrient Density, and Cost. Front. Nutr..

[5] Wang L., Martínez Steele E., Du M., Pomeranz J.L., O'Connor L.E., Herrick K.A., Luo H., Zhang X., Mozaffarian D., Zhang F.F. (2021). Trends in Consumption of Ultraprocessed Foods Among US Youths Aged 2–19 Years, 1999–2018. JAMA.

[6] Martínez Steele E., Baraldi L.G., Louzada M.L., Moubarac J.C., Mozaffarian D., Monteiro C.A. (2016). Ultra-processed foods and added sugars in the US diet: Evidence from a nationally representative cross-sectional study. BMJ Open.

[7] U.S Department of Agriculture and U.S. Department of Health and Human Services. Dietary Guidelines for Americans, 2020–2025..

[8] de Araújo T.P., de Moraes M.M., Afonso C., Santos C., Rodrigues S.S.P. (2022). Food Processing: Comparison of Different Food Classification Systems. Nutrients.

[9] Sadler C.R., Grassby T., Hart K., Raats M., Sokolovic M., Timotijevic L. (2021). Processed food classification: Conceptualization and challenges. Trends Food Sci. Technol..

[10] Martinez-Perez C., San-Cristobal R., Guallar-Castillon P., Martínez-González M., Salas-Salvadó J., Corella D., Castañer O., Martinez J.A., Alonso-Gómez Á., Wärnberg J. (2021). Use of Different Food Classification Systems to Assess the Association between Ultra-Processed Food Consumption and Cardiometabolic Health in an Elderly Population with Metabolic Syndrome (PREDIMED-Plus Cohort). Nutrients.

[11] Petrus R.R., Sobral P.J., Tadini C.C., Gonclaves C.B. (2021). The NOVA classification system: A critical perspective in food science. Trends Food Sci. Technol..

[12] Monteiro C.A. (2009). Nutrition and health. The issue is not food, nor nutrients, so much as processing. Public Health Nutr..

[13] Monteiro C.A., Cannon G., Levy R.B., Moubarac J.C., Louzada M.L.C., Pereira-Machado P. (2019). Ultra-processed Foods, Diet Quality, and Health Using the NOVA Classification System.

[14] Capozzi F., Magkos F., Fava F., Milani G.P., Agostoni C., Astrup A., Saguy I.S. (2021). A Multidisciplinary Perspective of Ultra-Processed Foods and Associated Food Processing Technologies: A View of the Sustainable Road Ahead. Nutrients.

[15] Martinez-Steele E., Khandpur N., Batis C., Bes-Rastrollo M., Bonaccio M., Cediel G., Huybrechts I., Juul F., Levy R.B., da Costa Louzada M.L. (2023). Best practices for applying the Nova food classification system. Nat. Food.

[16] Mambrini S.P., Menichetti F., Ravella S., Pellizzari M., De Amicis R., Foppiani A., Battezzati A., Bertoli S., Leone A. (2023). Ultra-Processed Food Consumption and Incidence of Obesity and Cardiometabolic Risk Factors in Adults: A Systematic Review of Prospective Studies. Nutrients.

[17] Menichetti G., Ravandi B., Mozaffarian D., Barabási A.L. (2023). Machine learning prediction of the degree of food processing. Nat. Commun..

[18] Poti J.M., Braga B., Qin B. (2017). Ultra-processed Food Intake and Obesity: What Really Matters for Health-Processing or Nutrient Content?. Curr. Obes. Rep..

[19] Zinöcker M.K., Lindseth I.A. (2018). The Western Diet-Microbiome-Host Interaction and Its Role in Metabolic Disease. Nutrients.

[20] Askari M., Heshmati J., Shahinfar H., Tripathi N., Daneshzad E. (2020). Ultra-processed food and the risk of overweight and obesity: A systematic review and meta-analysis of observational studies. Int. J. Obes..

[21] De Amicis R., Mambrini S.P., Pellizzari M., Foppiani A., Bertoli S., Battezzati A., Leone A. (2022). Ultra-processed foods and obesity and adiposity parameters among children and adolescents: A systematic review. Eur. J. Nutr..

[22] Moradi S., Entezari M.H., Mohammadi H., Jayedi A., Lazaridi A.V., Kermani M.A.H., Miraghajani M. (2023). Ultra-processed food consumption and adult obesity risk: A systematic review and dose-response meta-analysis. Crit. Rev. Food Sci. Nutr..

[23] Silva F.M., Giatti L., de Figueiredo R.C., Molina M.D.C.B., de Oliveira Cardoso L., Duncan B.B., Barreto S.M. (2018). Consumption of ultra-processed food and obesity: Cross sectional results from the Brazilian Longitudinal Study of Adult Health (ELSA-Brasil) cohort (2008-2010). Public Health Nutr..

[24] Beslay M., Srour B., Méjean C., Allès B., Fiolet T., Debras C., Chazelas E., Deschasaux M., Wendeu-Foyet M.G., Hercberg S. (2020). Ultra-processed food intake in association with BMI change and risk of overweight and obesity: A prospective analysis of the French NutriNet-Santé cohort. PLoS Med..

[25] Hall K.D., Ayuketah A., Brychta R., Cai H., Cassimatis T., Chen K.Y., Chung S.T., Costa E., Courville A., Darcey V. (2019). Ultra-Processed Diets Cause Excess Calorie Intake and Weight Gain: An Inpatient Randomized Controlled Trial of Ad Libitum Food Intake. Cell Metab..

[26] Forde C.G. (2023). Beyond ultra-processed: Considering the future role of food processing in human health. Proc. Nutr. Soc..

[27] Monteiro C.A., Moubarac J.C., Levy R.B., Canella D.S., Louzada M.L.D.C., Cannon G. (2018). Household availability of ultra-processed foods and obesity in nineteen European countries. Public Health Nutr..

[28] De Siena M., Raoul P., Costantini L., Scarpellini E., Cintoni M., Gasbarrini A., Rinninella E., Mele M.C. (2022). Food Emulsifiers and Metabolic Syndrome: The Role of the Gut Microbiota. Foods.

[29] Vo T.D., Lynch B.S., Roberts A. (2019). Dietary Exposures to Common Emulsifiers and Their Impact on the Gut Microbiota: Is There a Cause for Concern?. Compr. Rev. Food Sci. Food Saf..

[30] Baraldi L.G., Martinez Steele E., Canella D.S., Monteiro C.A. (2018). Consumption of ultra-processed foods and associated sociodemographic factors in the USA between 2007 and 2012: Evidence from a nationally representative cross-sectional study. BMJ Open.

[31] Marino M., Puppo F., Del Bo' C., Vinelli V., Riso P., Porrini M., Martini D. (2021). A Systematic Review of Worldwide Consumption of Ultra-Processed Foods: Findings and Criticisms. Nutrients.

[32] Tobias D.K., Hall K.D. (2021). Eliminate or reformulate ultra-processed foods? Biological mechanisms matter. Cell Metab..

[33] Juul F., Parekh N., Martinez-Steele E., Monteiro C.A., Chang V.W. (2022). Ultra-processed food consumption among US adults from 2001 to 2018. Am. J. Clin. Nutr..

[34] Aune D., Keum N., Giovannucci E., Fadnes L.T., Boffetta P., Greenwood D.C., Tonstad S., Vatten L.J., Riboli E., Norat T. (2016). Whole grain consumption and risk of cardiovascular disease, cancer, and all cause and cause specific mortality: Systematic review and dose-response meta-analysis of prospective studies. BMJ.

[35] Williams P.G. (2012). Evaluation of the evidence between consumption of refined grains and health outcomes. Nutr. Rev..

[36] Schwingshackl L., Bogensberger B., Hoffmann G. (2018). Diet Quality as Assessed by the Healthy Eating Index, Alternate Healthy Eating Index, Dietary Approaches to Stop Hypertension Score, and Health Outcomes: An Updated Systematic Review and Meta-Analysis of Cohort Studies. J. Acad. Nutr. Diet..

[37] Ong A.D., Uchino B.N., Wethington E. (2016). Loneliness and Health in Older Adults: A Mini-Review and Synthesis. Gerontology.

[38] Shan Z., Rehm C.D., Rogers G., Ruan M., Wang D.D., Hu F.B., Mozaffarian D., Zhang F.F., Bhupathiraju S.N. (2019). Trends in Dietary Carbohydrate, Protein, and Fat Intake and Diet Quality Among US Adults, 1999-2016. JAMA.

[39] Scrinis G., Monteiro C.A. (2018). Ultra-processed foods and the limits of product reformulation. Public Health Nutr..

[40] Adams J., Hofman K., Moubarac J.C., Thow A.M. (2020). Public health response to ultra-processed food and drinks. BMJ.

[41] Milley M.A., Forrest Faison C., Dana M.G., O’keefe G.B., Ediger M.A. (2017). Nutrition and Menu Standards for Human Performance Optimization.

[42] Sotelo-Díaz I., Blanco-Lizarazo C.M. (2019). A systematic review of the nutritional implications of military rations. Nutr. Health.

[43] McClung H.L., Armstrong N.J., Hennigar S.R., Staab J.S., Montain S.J., Karl J.P. (2020). Randomized Trial Comparing Consumption of Military Rations to Usual Intake for 21 Consecutive Days: Nutrient Adequacy and Indicators of Health Status. J. Acad. Nutr. Diet..

[44] Dixon A. (2013). MRE Production for 2014 Does Away With Lasagna, Refried Beans, and Fajitas. NCO J..

